# Inter-rater reliability assessment of antibiotic prescription quality by infectious diseases physicians, fellows, and pharmacists

**DOI:** 10.1017/ash.2023.509

**Published:** 2023-12-06

**Authors:** Rachel Bystritsky, Katherine Gruenberg, Emily Abdoler, Alexandra Hilts-Horeczko, Sarah B. Doernberg

**Affiliations:** 1 Division of Infectious Diseases, Department of Medicine, University of California San Francisco, San Francisco, CA, USA; 2 Department of Clinical Pharmacy, University of California San Francisco School of Pharmacy, San Francisco, CA, USA; 3 Department of Medicine, University of Michigan, Ann Arbor, MI, USA

## Background

Prospective audit and feedback (PAF), reviewing prescribed antimicrobials with subsequent feedback to prescribers, is a core antibiotic stewardship strategy associated with decreased rates of antimicrobial resistance and other clinical benefits.^1,2^ However, the optimal level and type of training required for reliable and accurate PAF is unclear. Additionally, what constitutes quality antibiotic prescribing, eg, whether the use is evidence-supported, can be subjective.

Prior studies investigating the reliability of expert retrospective reviews of abstracted antibiotic prescription data report wide variability in inter-rater reliability (IRR) (κ = 0.01–0.72).^3–7^ How the reliability of these assessments performs under realistic conditions is unknown and could inform best practices for PAF. We aimed to investigate the IRR of assessments regarding whether antimicrobial use is evidence-supported (eg, supported by available clinical data and concordant with evidence-based practice) under conditions mimicking PAF and to determine factors influencing IRR.

## Methods

We conducted a cross-sectional, observational study measuring IRR of antimicrobial prescriptions by health professionals at the University of California San Francisco (UCSF) Medical Center. One investigator selected ten adult patients representing a variety of antibiotic uses and admitting services from a list of patients prescribed broad-spectrum antimicrobials.

Invited participants included infectious diseases (ID) attending physicians, senior fellows, and ID pharmacists. Participants reviewed the EMR within a specified 8-hour period to evaluate whether prescribed antibiotics were supported by clinical data and evidence-based practice. Participants then rated each antibiotic regimen as evidence-supported or not, categorized therapy as empirical or definitive, and described challenges with assessments via an online questionnaire (Qualtrics, Provo, UT). Participants could use guidelines or other resources but were not instructed on a specific approach.

For assessment of overall agreement, we calculated IRR with a Fleiss’ kappa statistic. For evaluation of per-subject agreement, we calculated binomial proportions with confidence intervals. All quantitative calculations were computed using STATA version 15.0 (College Station, TX).^8^

Open-ended responses were evaluated through thematic analysis.^9^ Two investigators independently reviewed all responses and generated codes, met to compare codes, and developed a codebook. The investigators then re-coded each response, reconciled differences, and developed themes. The study was approved by the UCSF Institutional Review Board.

## Results

Thirteen out of 29 (44.8%) of ID physicians (*n* = 5), pharmacists (*N* = 3), and fellow (*N* = 5) participated. Six (46.2%) participants had prior PAF experience. For individual cases, the percent of raters assessing antimicrobials as evidence-supported ranged from 1/13 (7.7%) to 13/13 (100%). Overall agreement was fair (κ = 0.27, (95% confidence interval (CI): 0.01–0.51). IRR was higher among physicians (κ = 0.35, (95% CI: 0.14–0.51)) than pharmacists (κ = −0.07, (95% CI: −0.39 to 0.07)) and highest among fellows (κ = 0.46, (95% CI: 0.07–0.62)). Those lacking PAF experience showed greater agreement (κ = 0.30, (95% CI: 0.01–0.38)) compared to those with experience (κ = 0.15, (95% CI: −0.03 to 0.29)). Agreement was higher for patients receiving definitive (κ = 0.53, (95% CI: 0.14–0.88) versus empirical therapy (κ = 0.12, (95% CI: 0.04–0.15)) and those with positive (κ = 0.45, (95% CI: 0.27–0.62)) compared to negative microbiology (κ = 0.14, (95% CI: −0.01 to 0.21)). Per-subject agreement is shown in Table 1. Qualitative analysis of participants’ responses revealed four themes around challenging assessments: question of true infection, lack of knowledge, missing chart documentation, and case complexity (Supplementary Table).

**Table 1.** Case characteristics and agreement scores

Case #	% of respondents assessing regimen as evidence-supported	IC	+ Micro	Syndrome	Antibiotic	Day of therapy	Definitive vs empiric treatment	Service	Proportion agreement (CI)
**1**	**100%**	No	Yes	Cystic fibrosis exacerbation	MEM	1	Definitive	Medicine	1 (0.75, 1.00)
**2**	**7.7%**	Yes	Yes	Positive blood culture	VAN	4	Definitive	Malignant Hematology	0 .92 (0.64, 0.99)
**3**	**15.4%**	No	No	Drain prophylaxis	VAN	2	Empirical	Orthopedics	0.84 (0.54, 0.98)
**4**	**15.4%**	No	No	Hepatic encephalopathy	FEP+ MTZ	2 2	Empirical	Liver Transplant	0.84 (0.54, 0.98)
**5**	**76.9%**	No	No	Cellulitis	VAN	2	Empirical	Medicine	0.77 (0.46, 0.95)
**6**	**23.1%**	Yes	Yes	Complex SSTI	VAN+ MEM	3 10	Empirical	Malignant Hematology	0.77 (0.46, 0.95)
**7**	**30.8%**	Yes	No	Leukocytosis	FEP	3	Empirical	Medicine	0.69 (0.39, 0.91)
**8**	**46.2%**	No	Yes	Bronchiectasis exacerbation	C/T	15	Definitive	Advanced Lung Disease	0.53 (0.25, 0.81)
**9**	**46.2%**	No	No	Appendicitis	ETP	2	Empirical	General Surgery	0.53 (0.25, 0.81)
**10**	**53.8%**	No	No	Pneumonia	FEP	2	Empirical	Medicine	0.53 (0.25, 0.81)

Note. AZM, Azithromycin; FEP, Cefepime; C/T, Ceftolozane/tazobactam; ETP, Ertapenem; IC, Immunocompromised; MEM, Meropenem; MTZ, Metronidazole; SSTI, Skin and soft tissue infection; VAN, Vancomycin.

## Discussion

We found fair overall agreement among participants within the range of IRR previously reported for experts’ antimicrobial judgments.^4,6,7^ Full consensus was rare in our study. One prior study evaluating IRR of experts’ antibiotic assessments found poor initial IRR (κ = 0.01) improved after discussion (κ = 0.34) and uniform application of guidelines (κ = 0.74).^3^ Implementing collaborative, guideline-based processes in PAF may improve the reliability of these assessments, particularly when evidence gaps exist.

We also noted higher IRR among physicians compared to pharmacists. These findings contrast with a prior study reporting similar IRR between internal medicine physicians (κ = 0.75) and hospital pharmacists (κ = 0.82) evaluating antimicrobials for guideline adherence.^7^ ID fellows and those without prior PAF experience also demonstrated higher agreement in our study. These findings also differ from a prior study reporting similar agreement between residents and specialists, regardless of experience.^6^ In both studies, participants received explicit instructions for interpreting, whereas our study withheld guidance. These studies suggest instructions for interpretation of what constitutes “evidence-supported” therapy may improve IRR. Participants in our study also evaluated cases based on live information in the EMR, whereas those in prior studies made their evaluations based on abstracted case vignettes. The dynamic nature of an EMR may have increased the complexity of assessments compared to a well-described vignette.

We note several limitations. Patients in this study were admitted with complex medical problems to an academic tertiary care center, which may limit external validity to other hospitals. Additionally, the convenience sample of 13 experts reviewing 10 cases may limit the representativeness of our findings. As the sample size of reviewers was small, one or two reviewers deviating from the assessment of the rest of the group would significantly affect overall agreement. This study evaluated agreement, not accuracy of decisions. Ideally, accuracy would be measured against a gold standard, but this is challenging under real-world conditions with uncertain diagnoses. Lastly, although assessments were conducted within the same 8-hour timeframe, they were not truly simultaneous, and some reviewers may have had more information than others based on the time of review within that window.

In the end, evaluation of quality, rather than quantity, of antibiotic prescriptions is a subjective endeavor. Further work is needed to address the challenge of how to standardize and optimize real-world antimicrobial prescription reviews.

## Supporting information

Bystritsky et al. supplementary material Bystritsky et al. supplementary material

## Supplementary material

For supplementary material accompanying this paper visit http://doi.org/10.1017/ash.2023.509. click here to view supplementary material
