# Supplementary material for: Inter-rater reliability assessment of antibiotic prescription quality by infectious diseases physicians, fellows, and pharmacists
Source: Antimicrob Steward Healthc Epidemiol. 2023 Dec 6;3(1):e227. doi: 10.1017/ash.2023.509 (PMC10753503; doi:10.1017/ash.2023.509)
Supplement: Bystritsky et al. supplementary material [file S2732494X23005090sup001.docx]

Supplementary Table 1. Qualitative Themes and Examples of Representative Quotes

| **Theme** | **Illustrative Quotes** |
| --- | --- |
| Lack of knowledge | “…I actually don't know the data (if it exists) about the benefit for prophylactic antibiotics around spinal surgery if drains are left in place” (Respondent 1, Case 2)  “I don't manage uncomplicated appendicitis frequently so looked up the guidelines” (Respondent 2, Case 7)  “Had to look up Corynebacterium striatum literature susceptibilities” (Respondent 10, Case 9) |
| Missing chart documentation | “Want to confirm repeat cultures were drawn prior to antibiotics” (Respondent 7, Case 3)  “Didn’t have easy access to outside records…” (Respondent 3, Case 6)  “Notes quite sparse, difficult to evaluate for suspected spontaneous bacterial peritonitis or other infection” (Respondent 4, Case 10) |
| Question of true infection | “Hard to know how much of her disease is infection versus underlying lung disease” (Respondent 3, Case 1)  “Unable to interview patient for symptoms, which is crucial to determine if cystic fibrosis exacerbation requiring antibiotics (as no imaging evidence of pneumonia)”  (Respondent 12, Case 5)  “It is difficult to determine if the etiology of the patient's fevers/systemic inflammatory response syndrome is infectious or non-infectious from the chart.” (Respondent 8, Case 8) |
| Case complexity | “She also a complex prior history of *Staphylococcus* pneumonia at the outside hospital and microbiology with *Serratia* in the past. Needed some extra time to feel confident.” (Respondent 7, Case 6)  “…her rapidly changing clinical course made this a difficult case to review” (Respondent 8, Case 7)  “Immunocompromised host makes it slightly more challenging” (Respondent 2, Case 9) |
